# Are single mothers’ higher smoking rates mediated by dysfunctional coping styles?

**DOI:** 10.1186/1472-6874-14-124

**Published:** 2014-10-08

**Authors:** Stefanie Sperlich, Mercy Nyambura Maina

**Affiliations:** Medical Sociology, Hannover Medical School, Carl-Neuberg-Str. 1, 30625 Hannover, Germany

## Abstract

**Background:**

Smoking prevalence has been shown to be considerably higher among single mothers as compared to their married or cohabiting counterparts. This study examines whether this could be attributed to single mothers’ different capability in dealing with stress.

**Methods:**

Based on cross-sectional data of 3129 German mothers, the study explores the associations between single motherhood, coping styles and moderate and heavy smoking pattern using a regression-based ‘parallel multiple mediator model’.

**Results:**

Single mothers showed higher rates of negative coping styles than partnered mothers, holding for ‘self-blame/rumination’ (p < 0.001), ‘blaming others’ (p = 0.048) and in particular for ‘substance consumption’ (p < 0.001). With respect to positive coping styles the findings were heterogeneous: while partnered mothers scored higher on ‘active influence’ (p < 0.001), single mothers showed higher values of ‘positive self-verbalisation’ (p < 0.001). Evidence for a mediating effect of coping styles on the relationship between single motherhood and moderate as well as heavy smoking was only found for ‘substance consumption’. Moreover, single motherhood may moderate the effect of ‘self-blame/rumination’ on heavy smoking (p = 0.025). Against expectations, higher levels of ‘active influence’ were not associated with lower but with significant higher odds of moderate smoking (OR = 1.19).

**Conclusion:**

Single mothers compared to partnered mothers showed a different ability to cope with stress. However, only the coping strategy ‘substance consumption’ mediates the relationship between single motherhood and smoking. Exclusively in single mothers, ‘self-blame/rumination’ was associated with heavy smoking, indicating that they might utilize smoking as a way to come to terms with negative ruminative thoughts.

## Background

A wealth of empirical studies demonstrates increased smoking rates in single mothers compared with married and cohabitating mothers [1–4]. This phenomenon has been attributed to single mothers’ increased levels of psychosocial stress which could be found for financial hardship [5], poor psychosocial working conditions and work-family conflicts [6], child care responsibilities [2] and social exclusion [7]. According to the transactional model, stress results from an imbalance between demands and resources. This occurs when pressure exceeds one’s perceived ability to cope [8]. Coping can be defined as behaviours and thoughts of individuals who try to manage demands from the environment. In general, coping may be understood as predominantly adaptive or maladaptive. Problem-focused active coping is thought to be more likely to lead to positive outcomes than emotion-focused coping, which relies more on emotional venting and problem avoidance [9]. The stress-coping model of addiction [10] suggests that in the absence of more effective coping strategies, individuals use smoking as a means of coping with stress. Qualitative studies among low-income women confirm this assumption indicating that smoking is used as a way of coping with difficult and stressful circumstances in order to facilitate mood regulation [11–13]. The stress-coping model illustrates that both, perceived stress as well as coping resources, may be pertinent to vulnerability to tobacco use. First, those who report more perceived stress may be at heightened vulnerability to engage in smoking. Second, the manner in which individuals cope with stress can potentially buffer the perceived impact of that stress. The question whether single mothers are not only more exposed to stressors but also show a different ability to cope with stress has not been sufficiently explored so far. Previous research has revealed inconsistent results. Cohen & Dekel [13] found out that divorced mothers used less active coping and were less inclined to request advice and emotional support from friends and relatives than their married counterparts. In contrast, Avison et al. [14] found that single mothers did not react differently to stressors, and were not more vulnerable to them. Sachs et al. [15] found that the usual emotional coping strategies among low income single mothers were social isolation, downward social comparison, conflict avoidance but also self-reliance. Compas & Williams [16] observed that single compared to married mothers used more coping strategies related to accepting responsibility and positive reappraisal. As previous studies on coping strategies among single mothers are predominantly based on qualitative data with small sample sizes, their epidemiological significance may be limited. Hence, there is still a lack of knowledge to what extent single and partnered mothers differ with respect to coping strategies and how these differences may contribute to higher smoking rates in single mothers. Against this background, the aim of this work was to analyse the role of stress-related coping strategies in mediating the relationship between single motherhood and smoking patterns.

## Methods

### Sample

The sample consists of 3129 mothers with underage children (0–18 years of age) living in Germany. The ethics committee of Hannover Medical School approved the study. Data were collected in 2009 by means of mail survey. Single mothers are defined as mothers with at least one underage child living in the household without a partner, i.e. mothers cohabiting with a partner were not defined as single mothers but as ‘partnered mothers’. The sample was selected in proportion to the distributions of German federal states, school education, family status, mother’s age, and number of children. Subsequently, the cases were weighted in order to obtain data representative for German mothers. The final sample consisted of 3129 mothers with 506 (16.8%) out of them were single mothers. The sample characteristics and smoking prevalence of single and partnered mothers have been reported elsewhere [17]. It was pointed out that compared to partnered mothers (mean age 39.2 ± 6.5), single mothers (mean age 38.6 ± 6.9) were more likely to be full-time employed and in particular more frequently at risk of poverty.

### Smoking prevalence

Smoking pattern was assessed by asking the women whether they currently smoke cigarettes. Pipe smoking was not assessed. The outcome variable ‘current smoking status’ included three categories: (1) smoking daily (2) smoking occasionally (not daily) and (3) not smoking at all. If the women answered ‘smoking daily’ , they were asked how many cigarettes they smoked per day. In line with Billings and Moos [18] we selected 20 cigarettes per day as a level at which tobacco dependence was probable and used this as a threshold for heavy smoking. The ‘smoking pattern’ was then defined as a three category outcome variable with non-smoking (currently not smoking at all), moderate (1–19 cig./day) and heavy smoking (≥20 cig./day). A previous study on the same database illustrated that nearly every fifth single mother (19.2%) smoked at least 20 cigarettes a day, whereas this applied only to 8.4% of partnered mothers. Also moderate smoking patterns (24.8% versus 17.7%) as well as occasional smoking rates (6.8% vs. 5.0%) were higher in single than in partnered mothers [17].

### Coping styles

Coping styles were assessed using the INCOPE questionnaire with 14 items measuring positive and negative coping styles [19]. Positive coping styles were divided into two dimensions: 1. ‘positive self-verbalisation’ (three items, e.g. “I am reducing my distress by encouraging myself”) and 2. ‘active influence’ (three items, e.g. “I attempt to tackle and solve the problem”). Negative coping skills were assessed with three dimensions: 1. ‘self-blame/rumination’ (three items, e.g. “I ruminate for a long time and keep on thinking about the occurrence”), 2. ‘blaming others’ (three items, e.g. “I make other reproaches”) and 3. ‘substance consumption’ (two items, e.g. “I smoke, drink alcohol, eat sweets, or take tranquiliser in order to calm down”). The subjects were asked to rate how well each of the statements describe their general attitude or behavior in a stressful situation on a scale of 1 (never) to 5 (very often). Accordingly, each subscale has a sum score between 3 and 15 with the exception of ‘substance consumption’ ranging from 2 to 10. With respect to the subscale ‘active influence’, two items were reversed in a way that high values indicate high expression of this coping pattern. The reliability (Cronbach’s Alpha) of the subscales is α = 0.65 for ‘active influence’ , 0.68 for ‘positive self-verbalisation’ , 0.70 for ‘self-blame/rumination’ , 0.75 for ‘blaming others’ and 0.89 for ‘substance consumption’.

### Statistical analysis

**Figure 1:**
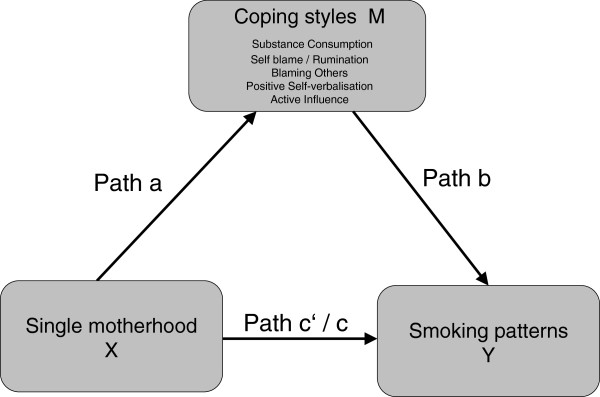
**The ‘Parallel Multiple Mediator Model’ of single motherhood on moderate and heavy smoking patterns.** Path c indicates the total, c’ the direct and path a * b the indirect effect of X on Y.

We used a ‘parallel multiple mediator model’ [20] in order to analyse the role of different coping styles on the relationship between single motherhood and smoking. According to Figure 1, coping styles (M) act as a mediator for the relationship between single motherhood (X) and smoking (Y), when X causally influences Y and M, and M causally influences Y. Since the strong association between X and Y (path c) has already been established on this database [17] we focus on the causal links between X and M (path a) as well as between M and Y (path b). Significance of path a was tested by means of ANOVA with single motherhood (yes versus no) as the independent variable and coping styles as the continuous dependent variables. Multinomial logistic regression analysis was performed for testing the significance of path b with coping styles as the continuous predictors and smoking patterns (moderate and heavy smoking versus non smoking) as the categorical outcomes. In order to answer the question whether the effect of coping styles on smoking holds for both single and partnered mothers, we displayed the results for both groups separately. For all analyses we adjusted for mothers’ age and age of youngest child.

In a second step, all paths of the ‘parallel multiple mediator model’ were examined simultaneously using the recently developed statistical tool PROCESS for SPSS and SAS [20]. By means of maximum likelihood logistic regression analysis we estimated the regression coefficients and their 95% confidence intervals of the total (path c, Figure 1) and direct effects (path c’ , Figure 1) of single motherhood on moderate and heavy smoking patterns as well as the indirect effects via coping styles (path a * b, Figure 1). The coping styles will only act as a mediator for the relationship between single motherhood and smoking if the effects of path a and b have the same algebraic signs (e.g. significant positive association between single motherhood and negative coping styles (path a) and significant positive association between negative coping styles and smoking (path b). The coping dimension ‘substance consumption’ was analysed separately from the other coping styles as there may be an overlap between the coping dimension ‘substance consumption’ and the outcome (smoking), although this coping dimension includes other substances as well. Inference about indirect effects in the mediation model was determined by 95% bias corrected bootstrap confidence intervals based on 1.000 bootstrap samples. If zero is not in the confidence interval, then the indirect effect is different from zero and can be considered as significant. As in some cases our findings pointed towards a possible *moderator*-effect of single motherhood, we additionally estimated two models with single motherhood as the moderator (M) on the relationship between coping styles (independent variable X) and smoking (dependent variable Y). Model 1 analysed the moderator-effect of single motherhood on the relationship between ‘self-blame/rumination’ and ‘heavy smoking’. Model 2 investigated the effect of single motherhood on the relationship between ‘active coping’ and ‘moderate smoking’. We calculated the coefficient for the product of the independent variable and the moderator (interaction term) and estimated the conditional effects of X on Y at each of the two values of the moderator, along with a standard error and p-value.

## Results

### Differences in coping styles between single and partnered mothers

Table 1 shows that negative coping strategies were more pronounced among single mothers. Compared to partnered mothers they scored higher on ‘self-blame/rumination’ (p = <0.001), ‘blaming others’ (p = 0.048) and in particular on ‘substance consumption’ (p = <0.001). With respect to positive coping styles the picture is mixed: Regarding ‘active influence’ , single mothers compared to partnered mothers showed a significantly lower mean value (p = <0.001) while the mean value of ‘positive self-verbalisation’ was significantly higher than in partnered mothers (p = <0.001).

**Table 1:** **Differences in coping styles between single and partnered mothers**

	Single mothers			Partnered mothers			ANOVA	
Coping styles	n	AM	SD	n	AM	SD	F	p value
**Substance consumption**	517	**2.99**	1.25	2579	**2.49**	1.22	72.86	**<0.001**
**Self-blame/rumination**	514	**3.10**	0.79	2566	**2.92**	0.78	22.63	**<0.001**
**Blaming others**	511	**2.22**	0.74	2570	**2.16**	0.68	3.90	**0.048**
**Positive self-verbalisation**	512	**3.23**	0.75	2536	**3.10**	0.74	12.98	**<0.001**
**Active influence**	507	**3.75**	0.66	2509	**3.86**	0.64	13.35	**<0.001**

Notes: AM = arithmetic mean value, SD = standard deviation, displayed are the mean values of the scales (ranging from 1 to 5). Bold values indicate significant effects.

### Impact of coping styles on smoking patterns among single and partnered mothers

As shown in Table 2, higher levels of ‘substance consumption’ were more frequently found among moderately and in particular among heavily smoking mothers as compared to non-smoking mothers. This association held for both partnered and single mothers, however, more pronounced among the latter, in particular with respect to heavy smoking. Only in single mothers, higher levels of ‘self-blame/rumination’ increased the odds of heavy smoking (OR = 1.24). Also exclusively in single mothers, higher levels of ‘active influence’ were associated with higher odds of moderate smoking (OR = 1.19). The coping styles ‘blaming others’ and ‘positive self-verbalisation’ showed neither an effect on moderate nor on heavy smoking. This held for single and for partnered mothers.

**Table 2:** **Impact of coping styles on moderate and heavy smoking pattern in single, partnered and all mothers**

	Single mothers (n = 442)				Partnered mothers (n = 2218)				All mothers (n = 2660)			
	Moderate (n = 115) vs. non-smoking (n = 236)		Heavy (n = 91) vs. non-smoking (n = 236)		Moderate (n = 413) vs. non-smoking (n = 1607)		Heavy (n = 198) vs. non-smoking (n = 1607)		Moderate (n = 528) vs. non-smoking (n = 1843)		Heavy (n = 289) vs. non-smoking (n = 1843)	
Coping styles	OR	CI 95%	OR	CI 95%	OR	CI 95%	OR	CI 95%	OR	CI 95%	OR	CI 95%
**Substance consumption**	**1.54**	**1.38-1.72**	**1.80**	**1.58-2.04**	**1.51**	**1.44-1.58**	**1.66**	**1.55-1.78**	**1.51**	**1.45-1.58**	**1.72**	**1.62-1.82**
**Self-blame/rumination**	1.04	0.93-1.16	**1.24**	**1.10-1.40**	0.98	0.93-1.04	1.00	0.94-1.07	0.99	0.95-1.04	1.06	1.00-1.12
**Blaming others**	1.07	0.95-1.20	0.94	0.84-1.06	0.96	0.90-1.02	0.99	0.91-1.07	0.99	0.94-1.04	0.97	0.91-1.03
**Positive self-verbalisation**	1.07	0.96-1.19	1.11	0.98-1.25	1.02	0.97-1.07	0.97	0.91-1.04	1.03	0.99-1.08	1.03	0.98-1.09
**Active influence**	**1.19**	**1.04-1.37**	1.08	0.94-1.25	1.03	0.97-1.10	1.03	0.94-1.13	1.05	0.99-1.11	1.02	0.95-1.09

Notes: adjusted for mothers’ age and age of youngest child, moderate smoking = < 20 cig./day, heavy smoking = ≥ 20 cig./day, OR = odds ratio, CI 95% = 95% confidence interval, significant effects in bold.

### Total, direct and indirect effects of single motherhood on smoking patterns

As can be seen in Table 3, the effect of single motherhood on moderate as well as heavy smoking decreased but remained significant after controlling for the coping style ‘substance consumption’ from c = 0.60 to c’ = 0.45 (moderate smoking) and from c = 1.15 to c’ = 0.97 (heavy smoking). The regression coefficients of 0.25 for moderate and 0.41 for heavy smoking pattern point towards a strong indirect effect in such a way that single mothers’ higher smoking rates could partly be attributed to higher values of the coping style ‘substance consumption’. All other coping styles which were analysed simultaneously showed no significant indirect effect on single mothers’ moderate or heavy smoking patterns. Most coping styles showed negative coefficients. This is due to the fact that path a and b (Figure 1) have opposite algebraic signs indicating that these coping styles do not fulfil the condition for mediation.

**Table 3:** **Total, direct and indirect effects of single motherhood (SM) on moderate and heavy smoking – results of the ‘mediation model’**

	Moderate smoking			Heavy smoking		
	Effect	SE	p	Effect	SE	p
**Total effect of SM**	**0.597**	**0.14**	**<0.001**	**1.147**	**0.15**	**<0.001**
**Direct effect of SM controlled for substance consumption**	**0.445**	**0.14**	**0.019**	**0.966**	**0.15**	**<0.001**
**Direct effect of SM controlled for other coping strategies**	**0.606**	**0.16**	**0.013**	**1.144**	**0.18**	**<0.001**
**Indirect effects**	**Effect**	**Boot SE**	**Boot 95 CI**	**Effect**	**Boot SE**	**Boot 95 CI**
**Substance consumption**	**0.253**	**0.06**	**0.14** – **0.37**	**0.413**	**0.08**	**0.24** – **0.57**
**Self-blame/rumination**	-0.001	0.01	-0.02 – 0.01	0.012	0.01	-0.01 – 0.06
**Blaming others**	-0.003	0.01	-0.03 – 0.00	-0.002	0.01	-0.03 – 0.01
**Positive self-verbalisation**	0.011	0.01	0.00 – 0.04	-0.003	0.01	-0.03 – 0.02
**Active influence**	-0.013	0.01	-0.02– 0.01	-0.004	0.02	-0.04 – 0.02

Notes: adjusted for mothers’ age and age of youngest child. SE = Standard error, p = probability, Boot SE = bootstrap standard error, Boot 95 CI = bootstrap 95% confidence intervals. SM = single motherhood, n = 2410 (moderate smoking), n = 2192 (heavy smoking), significant effects in bold.

### Conditional effects of coping styles on smoking

As Table 2 suggests, the effects of ‘self-blame/rumination’ on heavy smoking and of ‘active influence’ on moderate smoking are different for single and partnered mothers pointing towards a moderator-effect of single motherhood. Thus, we additionally estimated two moderation models. The results showed no significant interaction term between single motherhood and the coping style ‘active influence’ (p = 0.311). However, the interaction term between single motherhood and the coping style ‘self-blame/rumination’ revealed to be significant (p = 0.025) (data not shown). The test for the conditional effect illustrated that ‘self-blame/rumination’ was significantly associated with heavy smoking in single mothers (p = 0.024) while no effect was found among partnered mothers (p = 0.525) (Table 4).

**Table 4:** **Conditional effect of ‘self-blame/rumination’ on heavy smoking for the two values of family status – results of the ‘moderation model’**

Moderator: family status	Effect	SE	p
**Partnered mothers**	-0.021	0.03	0.525
**Single mothers**	**0.116**	**0.05**	**0.024**

Notes: adjusted for mothers’ age and age of youngest child. SE = Standard error, p = probability, significant effects in bold.

## Discussion

Our study revealed that single mothers more frequently reported that they consume something (cigarettes, alcohol, sweets, anti-depressants) in order to calm down in stressful situations. This lends empirical evidence to the assumption that in dealing with stress, single mothers are more likely to use a passive, emotion-focused coping strategy, which relies on emotional venting. Our findings demonstrate that single mothers scored also higher on the dysfunctional coping styles ‘blaming others’ and ‘self-blame/rumination’ indicating that they are more prone to repetitively focus on the symptoms of distress. This may suggest that single mothers are not only more exposed to stressors in their daily life but that they also have a different ability to cope with stress. This result contradicts the findings reported by Avison et al. [14] who could not find any evidence that single mothers were more vulnerable or reactive to stressors than their married counterparts. Possibly, single mothers’ different ability to cope with stress reflects their higher stress exposure which could be established for different stressors, such as financial hardship, poor psychosocial working conditions as well as work-family conflicts and social exclusion [5, 6, 17]. It is assumed that the level of perceived daily stress has an impact on the way of coping in such a way that the likelihood of dysfunctional coping strategies increases with levels of psychosocial stress. Hobfoll et al. [21] point in this direction by stressing that, following stressful circumstances, individuals have an increasingly depleted resource pool to combat further stress. This depletion impairs individuals’ capability of coping with further stress, thus resulting in a vicious circle. However, with respect to positive coping styles we found single mothers scoring lower on ‘active influence’ but higher on ‘positive self-verbalisation’. The latter result puts the findings into perspective and also draws attention to single mothers’ competences in dealing with stress. If confirmed in further studies, these adaptive coping strategies should be considered as available resources and given more attention in health promoting programs for single mothers.

### Effect of coping styles on smoking in single and partnered mothers

We found a positive relationship between the emotion focussed coping style ‘substance consumption’ and smoking indicating that the higher the women scored on this coping style the more they were likely to smoke. This association held for both single and partnered mothers, however revealed to be stronger in single mothers. Hence, it may be assumed that heavy smoking in single mothers is more strongly motivated by the need to calm down in stressful situations whereas heavy smoking in partnered mothers has to a lesser extent to do with stress. This finding supports the assumption that higher levels of perceived stress may account for higher smoking prevalence in single mothers.

We also found that heavily smoking single mothers scored higher on ‘self-blame/rumination’ whereas partnered mothers who smoked at least 20 cigarettes per day did not have significant higher levels of rumination. This result supports the assumption that single motherhood acts as a moderator on the relationship between rumination and heavy smoking. Lerman et al. [22] found that smokers who are prone to negative moods use tobacco consumption in order to reduce emotional upset. Our findings suggest that particularly heavily smoking single mothers utilize smoking as a means to come to terms with negative ruminative thoughts. Richmond et al. [23] emphasized that rumination and smoking can reinforce each other and create a vicious circle. They assumed that smoking also enhanced the risk of rumination as nicotine’s pharmacological effects support to focus more effectively on negative self-referential thoughts. Our study confirmed that ruminative thoughts and heavy smoking are closely connected in single mothers. This result underlines the relevance of cognitive-behavioural treatment programs in particular for heavily smoking single mothers in order to escape from the vicious circle of rumination and smoking.

Against expectation our findings revealed that single mothers scoring high on ‘active influence’ showed higher smoking rates, holding in particular for moderate smoking. This result is controversial because it opposes numerous studies suggesting that these strategies are adaptive and health promoting (e.g. [24]). However, previous studies found that when active coping was utilized in the presence of low socioeconomic resources the health-conducive effect of this coping style turned into its opposite and becomes detrimental to health. This was attributed to the fact that continuous high-effort coping with demanding psychosocial stressors exceeds personal coping resources as indexed by low socio-economic status [25, 26]. Our study indicates that this may also hold for some single mothers which might have implications for primary prevention programs addressing deficits in coping skills. Our findings suggest that if the source of single mothers’ distress is due to structural disadvantages, focusing solely on increasing active coping would not seem the proper strategy for health promotion.

Finally, our mediation analysis revealed that the coping style ‘substance consumption’ was the only powerful mediator on the relationship between single motherhood and smoking. However, it could also be the other way around in the sense that smoking leads to higher scores on ‘substance consumption’ , although this coping dimension includes other substances than smoking as well. Notwithstanding these limitations, there is some evidence that single mothers’ higher smoking rates may partly be attributed to their higher inclination to consume substances in order to calm down in stressful situations. Given that the ways of coping can only be properly judged by taking the level of perceived stress into account, further studies are needed in order to analyse the interplay between stress, coping and smoking behaviour.

## Conclusions

Single mothers as compared to their married or cohabiting counterparts showed higher rates of negative coping styles but in part also higher levels of positive coping strategies. Evidence for a mediating effect of coping styles on the relationship between single motherhood and smoking was found for ‘substance consumption’. In addition, single motherhood turned out to moderate the effect of ‘self-blame/rumination’ on heavy smoking. Contrary to expectation, high scores on ‘active influence’ were not accompanied by lower but by higher rates of moderate smoking in single mothers. All in all, our findings suggest that the different ability to cope with stress did not sufficiently explain single mothers’ higher smoking rates.

### Limitations of this study

We assumed that single mothers’ ways of coping predict smoking behaviour. However, firm conclusions cannot be drawn about the causal relationship between smoking and coping as the data used were cross-sectional. To analyse the causal relationship between coping and smoking, methodologically challenging longitudinal studies are needed that follow single mothers over a longer period of time. In addition, it has to be mentioned that the INCOPE questionnaire used for measuring functional and dysfunctional coping styles did not cover all possible dimensions of coping. Lastly, the questionnaire used in this study did not provide information about the smoking history of respondents and their past attempts to quit smoking.
